# Intrinsic transcriptomic sex differences in human endothelial cells at birth and in adults are associated with coronary artery disease targets

**DOI:** 10.1038/s41598-020-69451-8

**Published:** 2020-07-23

**Authors:** Robin J. G. Hartman, Daniek M. C. Kapteijn, Saskia Haitjema, Mireille N. Bekker, Michal Mokry, Gerard Pasterkamp, Mete Civelek, Hester M. den Ruijter

**Affiliations:** 1 Laboratory of Experimental Cardiology, University Medical Center Utrecht, Utrecht, The Netherlands; 2 Central Diagnostics Laboratory, University Medical Center Utrecht, Utrecht, The Netherlands; 3 Department of Obstetrics and Gynecology, University Medical Center Utrecht, Utrecht, The Netherlands; 4 Center for Public Health Genomics, Department of Biomedical Engineering, University of Virginia, Charlottesville, VA 22908 USA; 5 Division of Heart and Lungs, Department of Experimental Cardiology, University Medical Center Utrecht, Heidelberglaan 100, PO Box 85500, 3508GA Utrecht, The Netherlands

**Keywords:** Cell biology, Physiology, Cardiology

## Abstract

Sex differences in endothelial cell (EC) biology may reflect intrinsic differences driven by chromosomes or sex steroid exposure and gender differences accumulated over life. We analysed EC gene expression data from boy–girl twins at birth and in non-twin adults to detect sex differences at different stages of life, and show that 14–25% of the EC transcriptome is sex-biased. By combining data from both stages of life, we identified sex differences that are present at birth and maintained throughout life, and those that are acquired over life. Promisingly, we found that genes that present with an acquired sex difference in ECs are more likely to be targets of sex steroids. Annotating both gene sets with data from multiple genome-wide association studies (GWAS) revealed that genes with an intrinsic sex difference in ECs are enriched for coronary artery disease GWAS hits. This study underscores the need for treating sex as a biological variable.

## Introduction

Endothelial cells (ECs) are located at the innermost lining of all vessels and are responsible for circulatory homeostasis. ECs are implicated in a plethora of functions, such as water and nutrient transfer, inflammation, vascular tension, hemostasis, and angiogenesis^1^. Differences in EC biology between the sexes have been described, indicating that sex is an important variable in EC biology^2^. Studies focussing on functional differences, i.e. migration, proliferation, and shear stress response, imply sex differences in regulation of key endothelial pathways, such as the redox system^3–5^. Differences between female and male cells might be present from birth onwards, or acquired later in life because of sex steroid exposure and gender differences. Pinpointing master regulators herein has been difficult, as differences between the sexes may be caused by sex hormones and/or sex chromosomes, which are difficult to study separately or to dissect. Transcriptomic studies of female and male (endothelial) cells shed light on differentially expressed genes (DEGs) between the sexes and their regulators, but few have been performed thus far on ECs^4^. Tissue-specific comparisons have been made using publicly available data from the Genotype-Tissue Expression project^6,7^, showing the importance of looking at sex differences in transcriptomics, but it may be difficult to extrapolate this to individual cell-types.

We analysed transcriptomes of human ECs at birth of opposite sexed twins and at an adult stage to interrogate both intrinsic and acquired sex differences. We hypothesized that the sex chromosomes are mainly involved in intrinsic sex differences, i.e. consistently different across the lifespan, while sex steroids and gender differences are the main players for acquired sex differences in human ECs. As it has been shown that genes that present with sex differences in expression are more likely to be less evolutionary conserved in their gene bodies, while their promoter, and hence their regulation, is more likely to be evolutionary conserved^8–10^, we looked at their evolutionary conservation to see whether this is different for acquired and intrinsic sex differences. Finally, we determined whether or not the genes with sex differences in EC had associations with (disease) traits in the genome-wide association study (GWAS) catalog.

## Results

### Workflow of the study

**Figure 1:**
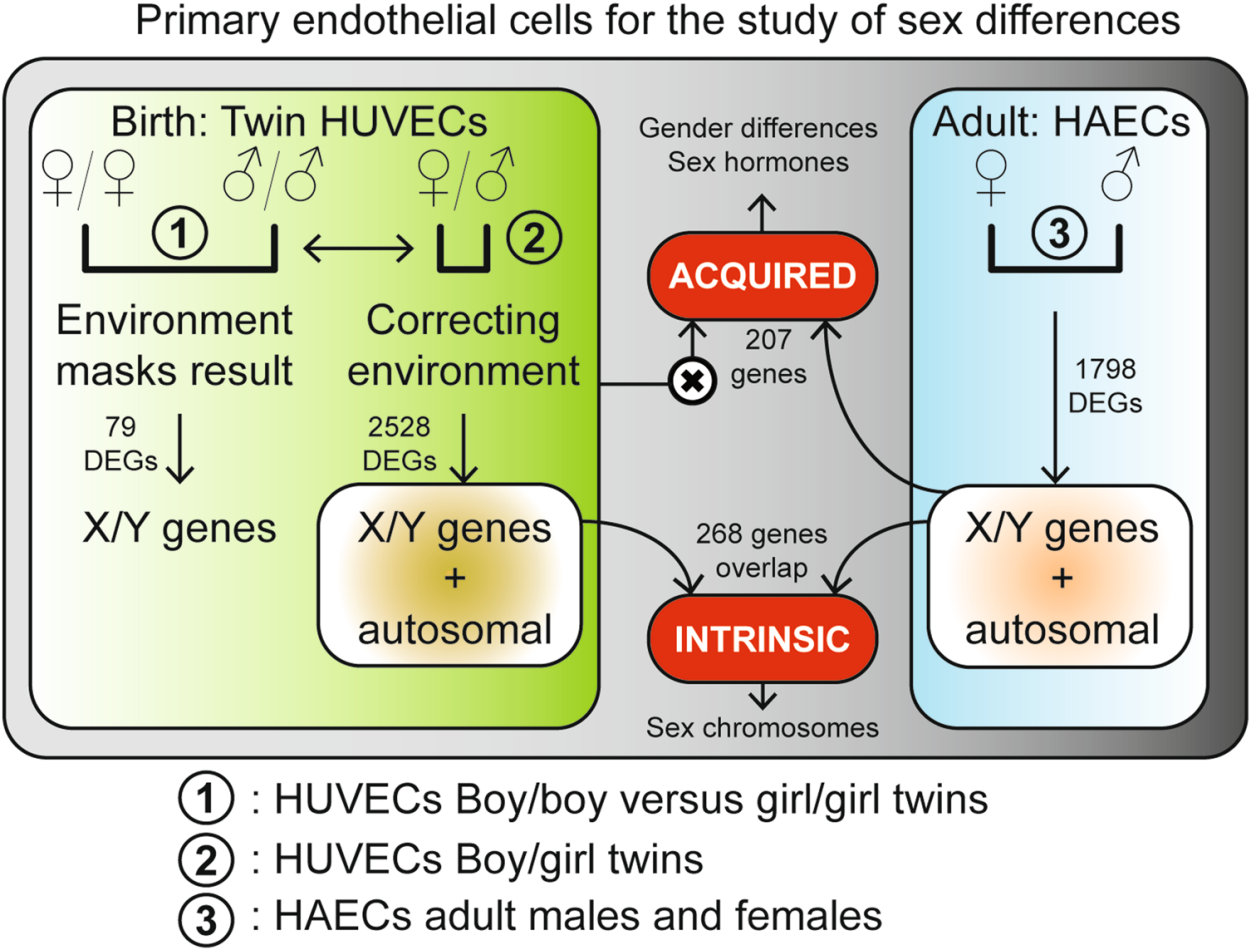
Workflow of the study. An overview of the analyses and comparisons used in the study is shown. The comparisons used in this study are numbered from 1–3. #1: HUVECs of boy–boy and girl–girl twins. #2: HUVECs of boy–girl twins. #3: HAECs of adult males and females. The amount of differentially expressed genes (DEGs) are shown as well as the definitions of the two different gene-sets (intrinsic and acquired).

We analysed sex differences in multiple settings (Fig. 1). We used human umbilical vein EC (HUVEC) RNA-sequencing data from twins to determine sex differences at birth (7 boy–girl twins, 3 boy–boy twins, 4 girl–girl twins), and cultured human aortic EC microarray data from adults to determine sex differences at adulthood (43 female and 129 male samples). From these comparisons we generated lists of genes with either an intrinsic or an acquired sex difference. We defined intrinsic sex differences as those genes differentially expressed at birth, but also concordantly differentially expressed in adult endothelium. We defined acquired sex differences as those genes with no differential expression in the boy–girl twins (*p-*value > 0.5) and differentially expressed in adults *(p*-value < 0.05).

### Sex differences in the endothelial transcriptome at birth

**Figure 2:**
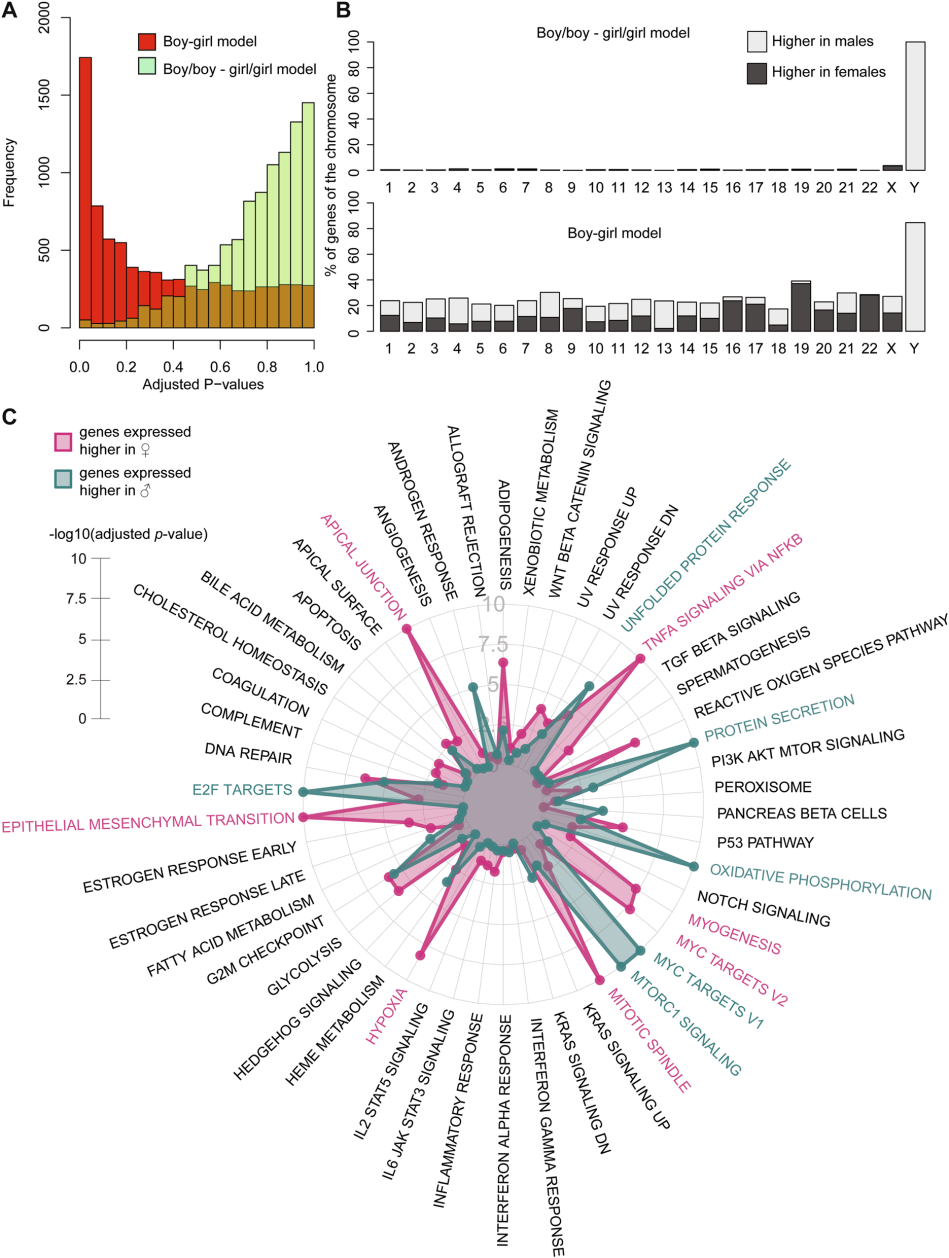
Sex differences in the endothelial transcriptome at birth. (**A**) Histograms of adjusted p-values are shown for both the boy–boy/girl–girl twins (green) and the boy–girl twins in which can be corrected for mother (red). (**B**) The number of differential genes per chromosome is shown in percentages of all genes interrogated on that chromosome (top for boy–boy/girl–girl twins, bottom for boy–girl twins). Lightgrey indicates higher expression in males (notice the Y-chromosome), darkgrey indicates higher expression in females. (**C**) Hallmark gene enrichments are shown in a radar plot. Blue highlights the significance of the gene enrichment of genes higher expressed in males, whereas pink shows this for genes higher expressed in females. The length of the radius measures significance (− log10 adjusted *p*-value).

The first step in our analysis was to analyse sex differences in the HUVEC transcriptome at birth. By using both boy–girl twins and boy–boy/girl–girl twins we could validate our own model, as it allowed us to correct for the microenvironment (mother) the boy–girl twins developed in. Boy–girl twin differences were more abundant as compared to the boy–boy twin and girl–girl twin comparison, as exemplified by the differential skewing of *p-*values in the groups of twins (Fig. 2A). In the comparison within the boy–girl twins, we identified 2,528 DEGs (25% of the interrogated transcriptome) between the sexes, of which 114 were on chromosome X or Y (4.5%, Fig. 2B). We detected 79 DEGs (FDR < 0.1) in the comparison boy–boy versus girl–girl twins of which 27 were located on the sex chromosomes (34.1% of the total number of DEGs, Fig. 2B). Gene hallmark analysis on DEGs at birth of the boy–girl twins higher expressed in girls showed enrichment of endothelial activation pathways such as endothelial to mesenchymal transition, hypoxia and NFkB-signaling (Fig. 2C, Suppl. Fig. 2). The genes that are significantly higher expressed in the boys of the boy–girl twins point to Myc targets, oxidative phosphorylation and mTOR signalling (Fig. 2C, Suppl. Fig. 2).

### Sex differences in the adult endothelium

**Figure 3:**
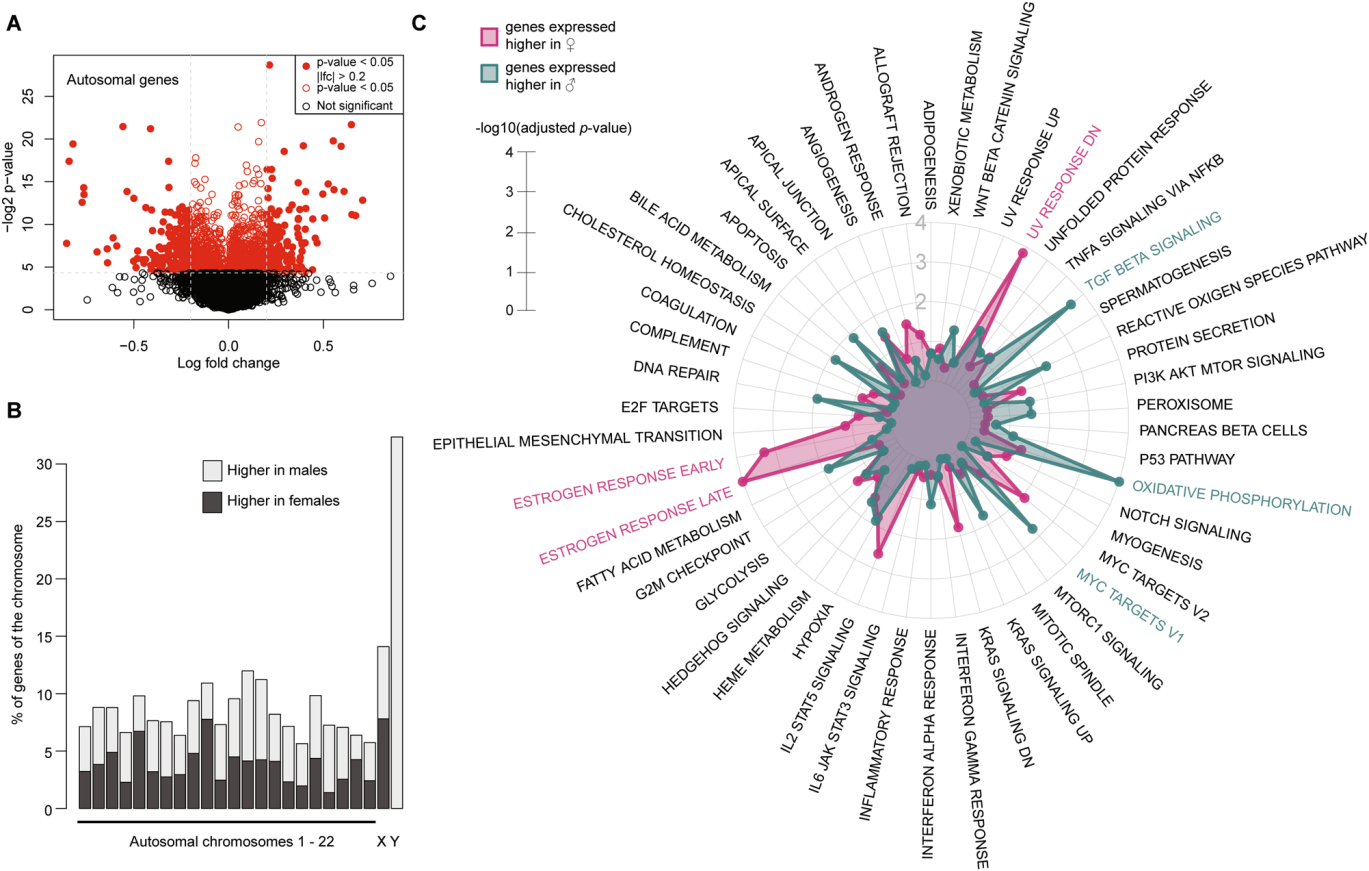
Sex differences in the adult endothelial transcriptome. (**A**) A volcano-plot is shown for all probes that map to non-duplicate autosomal genes. A complete one with sex chromosomal genes is shown in Suppl. Fig. 3A. Y-axis: – log2 p-value; x-axis: log fold change. (**B**) The amount of differential genes per chromosome between the sexes in the adult stage is shown in percentages of all genes interrogated on that chromosome. Lightgrey indicates higher expression in males (notice the Y-chromosome), darkgrey indicates higher expression in females. Only genes with an absolute log-fold change of higher than 0.2 are shown. (**C**) Hallmark gene enrichments are shown in a radar plot. Blue highlights the significance of the gene enrichment of genes higher expressed in males, whereas pink shows this for genes higher expressed in females. The length of the radius measures significance (− log10 adjusted *p*-value).

The second step of the analysis was to determine sex-differential DEGs in adult HAECs. Gene expression analysis of adult HAECS found 1,798 DEGs (*p* < 0.05, 14% of the interrogated transcriptome) between the sexes. Out of these 1,798 genes, 845 were higher expressed in males, and 953 were higher expressed in females (autosomal genes in Fig. 3A & all genes in Suppl. Fig. 3A). We found that 117 genes from the 1,798 were located on the sex chromosomes (6.5%, Fig. 3B), more than the ratio of sex chromosomal:autosomal genes on the microarray (4.4%), pointing to an enrichment of sex chromosomal genes. Gene hallmark enrichment on the genes higher expressed in adult female ECs points to estrogen responses, while genes higher expressed in the male cells show more oxidative phosphorylation pathways, as at birth, as well as Myc targets (Fig. 3C, Suppl. Fig. 3B). Upstream regulator analysis using the Ingenuity Pathway Analysis software also highlighted beta-estradiol (*p* = 7.69e−16) and Myc (*p* = 2.56e−12) as upstream regulators of the sex differences found in genes higher expressed in females or males, respectively (Suppl. Data File, log fold changes female over male).

### Intrinsic and acquired sex differences

**Figure 4:**
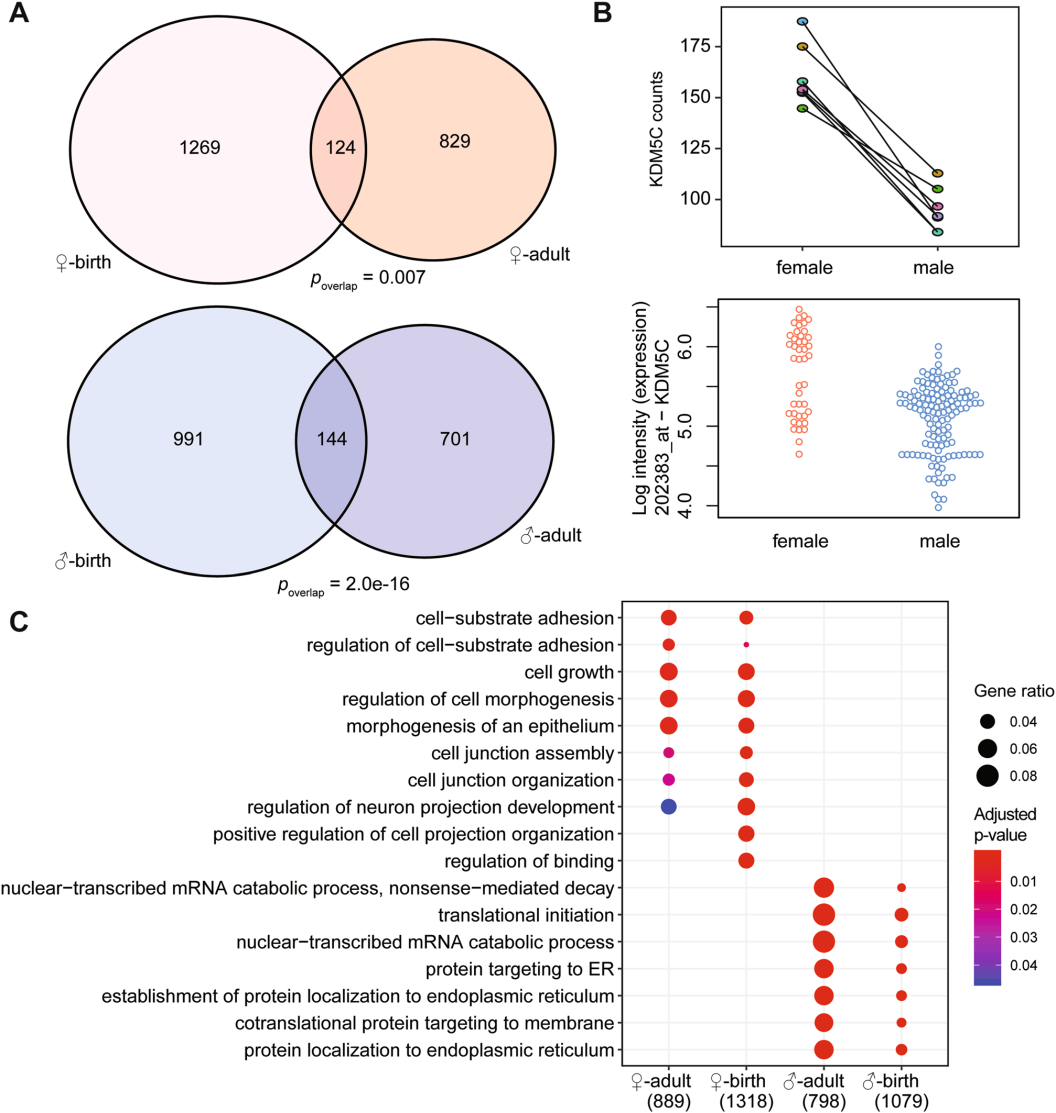
Comparing the sex differences in adults and at birth. (**A**) Venn-diagrams are shown for genes higher in males at birth and at the adult stage (blue circles), and for genes higher in females at birth and at the adult stage (pink circles). (**B**) *KDM5C* expression is plotted in the boy–girl twins (top) between males and females, and at the adult stage (bottom). (**C**) A dotplot is shown for Gene Ontology enrichments in the four different gene sets; genes higher in females in the adult stage, genes higher in females at birth, genes higher in males in the adult stage, and genes higher in males at birth. Terms are allocated to the rows, color indicates significance, and size of the dot indicates the ratio of genes from the set present. The number indicates the number of genes that could be found in any of the sets tested. The numbers of genes in each Gene Ontology term and overlaps are shown in Suppl. Fig. 4.

**Figure 5:**
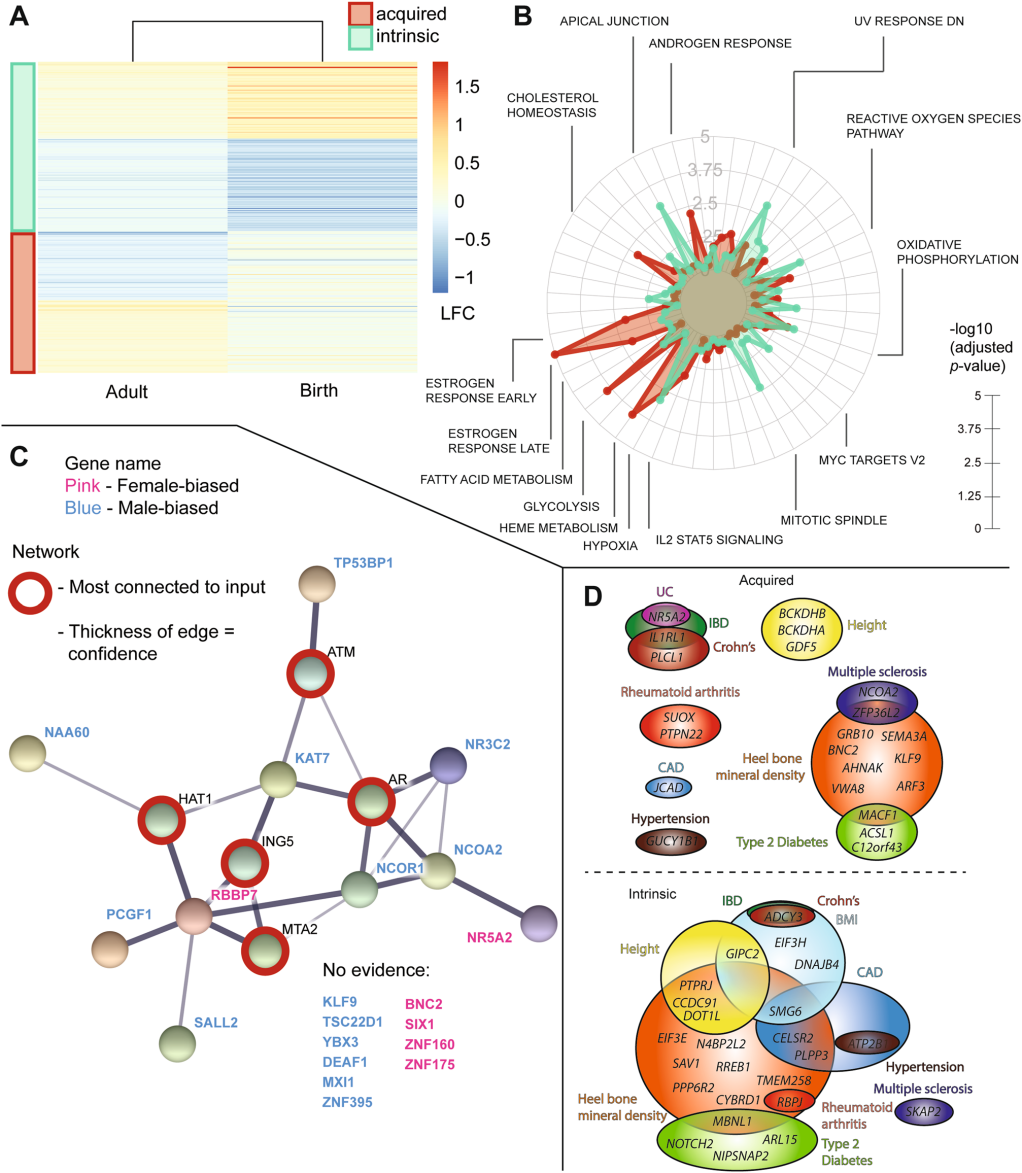
Comparing intrinsic and acquired sex differences. (**A**) A heatmap is drawn indicating the log-fold changes at birth and in the adult setting. A positive value means expression is higher in females. The intrinsic gene block is colored in green, the acquired gene block is colored in red. (**B**) Hallmark enrichments are shown in a radarplot in the same order as Figs. 2C and 3C, with green indicating intrinsic gene enrichment and red acquired gene enrichment. Interesting significant terms are highlighted. The length of the radius measures significance (− log10 adjusted *p*-value). (**C**) A StringDB network is shown of transcription factors and epigenetic modifiers in the acquired gene set. The top 5 most connected nodes to this input have been drawn as well and are marked by a red circle. Blue letters indicate higher expression of a gene in adult male HAECs, whereas pink letters indicate higher expression in females. The thickness of the edge indicates the confidence for the association as determined by the String analysis. (**D**) Two schematics are shown that highlight the genes of the different individual GWAS tested that overlap with either the acquired (top) or intrinsic (bottom) gene set. Colors indicate the GWAS, and the same colors are used for both panels. Acquired DEGs did not overlap with the BMI or the Alzheimer’s disease GWAS, while intrinsic DEGs did not overlap with the Alzheimer’s disease or the UC GWAS. *BMI* body mass index, *CAD* coronary artery disease, *Crohn’s* Crohn’s disease, *IBD* inflammatory bowel disease, *UC* ulcerative colitis.

In total, 268 genes were concordantly differentially expressed at birth and in adults and thus showed an intrinsic sex difference, of which 124 were higher in females (*p*_overlap_ = 0.007), and 144 in males (*p*_overlap_ = 2.0e−16, Fig. 4A). Of these 268 genes, 34 were sex-chromosomal (12.7%). An example, *KDM5C* (X-inactivation escapee), can be found in Fig. 4B, which is higher expressed in females at birth and during adulthood. Interestingly, 234 autosomal genes are intrinsically different between the sexes in the endothelium. Gene Ontology enrichments on genes that are higher expressed in females at birth and adult showed similarity, as well as those that are higher in males at birth and adult (Fig. 4C). However, the overlap between the genes contributing to these similar Gene Ontology enrichments at birth and in adults is low. E.g. for cell-substrate adhesion, 7 genes overlap of the 39 genes higher in females at birth and the 48 higher in adult female cells (Suppl. Fig. 4). We found 207 genes that fulfilled the criteria of our definition of an acquired sex difference, being differentially expressed in adults, but not at birth, of which 107 were higher expressed in females, and 100 in males (Fig. 5A, Suppl. Data File).

### Comparing intrinsic and acquired sex differences

As compared to the intrinsic sex differences, the acquired DEGs were less likely to be sex chromosomal (12 out of 207, 5.8%, only X-chromosomal). Previously, it has been shown that genes that present with sex differences in expression are more likely to be less evolutionary conserved in their gene bodies, while their promoter, and hence their regulation, is more likely to be evolutionary conserved^8–10^. We determined the evolutionary conservation of promoters and genes by calculating the PhastCon scores of the acquired and the intrinsic gene set. PhastCon scores tended to be higher for the promoters of genes with either an intrinsic or an acquired sex difference in expression (*p*_permutation_ over mean = 0.013–0.079), while the sequence of the gene itself had lower PhastCon scores (*p*_permutation_ over mean = 4e^−04^–0.013, Suppl. Fig. 5). High and low classification for PhastCon scores showed that promoters of genes with an acquired sex difference were more often in the class with high conservation as compared to random genes (*p*_permutation_ = 0.0228), while the sequence of genes with an intrinsic sex difference were more often in ‘low’ class (*p*_permutation_ = 0.0036, Suppl. Fig. 6).

### Gene hallmark enrichment

Even though the intrinsic and acquired sex differences gene lists are non-overlapping, both were enriched for hypoxia response and oxidative phosphorylation (Fig. 5B). The acquired DEGs, but not the intrinsic DEGs, were enriched for the sex-hormonal terms, such as estrogen and androgen response. The acquired DEGs are more likely to contain sex-hormonal targets than those with an intrinsic sex difference (Fisher’s Exact Test; *p* = 0.0002). The hallmarks for the genes with an intrinsic sex difference are “UV response” and “apical junction”.

### Protein associations to intrinsic and acquired regulatory sex differences

To determine potential associated regulators that would be missed by using transcriptomic data alone, we performed a String network analysis on gene expression regulators (transcription factors and epigenetic modifiers) in both the acquired and intrinsic sex difference gene-sets. The network for the regulatory genes with an acquired sex difference had significantly more interactions than expected (20 nodes, 7 edges, PPI-enrichment *p* = 0.029), which increased after expanding the network with the top 5 most connected associations to our 20 nodes (25 nodes, 22 edges, PPI-enrichment *p* = 0.000949). There were more transcription factors in the acquired gene set as compared to the intrinsic gene set (7.2% versus 5.2%), whereas epigenetic modifiers were more common in the intrinsic gene set (3.4% versus 6.3%) (Suppl. Fig. 7). The top 5 most connected associated proteins to the regulators of the acquired gene set were the androgen receptor (AR), ATM, HAT1, ING5, and MTA2 (Fig. 5C). The top 5 most connected associated proteins to the regulators of the intrinsic gene set all grouped together with only one of the entries (CDC5L, Suppl. Fig. 8), however, the network itself was already more connected without adding extra nodes (nodes: 25 versus 30, edges: 17 versus 34), indicating that it is a more complete network as compared to the network build from the acquired gene set.

### Associations of GWAS traits to intrinsic and acquired sex differences

To further describe the intrinsic and acquired gene sets, we determined whether or not genes that present with either an intrinsic or acquired sex difference in endothelial cells were targets in major genome-wide association studies (GWAS). We analysed GWAS for coronary artery disease (CAD)^11^, inflammatory bowel disease^12^, Crohn’s disease^12^, ulcerative colitis^12^, Alzheimer’s disease^13^, body mass index^14^, heel bone mineral density^15^, height^16^, hypertension^17^, multiple sclerosis^18^, rheumatoid arthritis^19^, and type 2 diabetes mellitus^20^. Specifics of the used GWAS studies can be found in Suppl. Data File. There was overlap between GWAS targets and either the acquired or intrinsic gene sets for all GWAS mentioned, except Alzheimer’s disease (see Fig. 5D). The acquired gene set was not significantly enriched for any GWAS as compared to random genes. However, the intrinsic gene set contained more genes from the CAD GWAS than randomly expected (p_permutation_ for more genes than expected = 0.0085, Suppl. Data File). These genes are *PLPP3*, *CELSR2*, *ATP2B1*, and *SMG6*. The intrinsic gene set also contained more genes that are targets in multiple GWAS, as compared to the acquired gene set (Fig. 5D).

We also determined whether or not intrinsic or acquired DEGs had an association with any trait in the GWAS catalog. Genes in the acquired set were more likely to have been mapped to by a common variant associated to a trait in the GWAS catalog than random genes (*p*_permutation_ = 0.01), while the intrinsic set is less likely mapped to as compared to random genes (*p*_permutation_ = 0.02, Suppl. Fig. 9). A data file with all the genes that show an intrinsic or an acquired sex difference and their associated information can be found in the Suppl. Data File.

## Discussion

In the present study, we show that, by using a unique twin model, sex differences in the EC transcriptome are profuse. We showed that a boy–girl twin model is superior to a boy–boy girl–girl twin model for detecting sex differences, since the boy–girl twins developed in the same microenvironment, whereas this is not the case for the comparison between boy–boy and girl–girl twins. Furthermore, by combining data from both stages of life, we identified sex differences that are present at birth and maintained throughout life, and those that are acquired over life. As we expected, acquired sex differences were enriched for hormonal responses, such as genes influenced by estrogens or androgens. Sex differences in transcriptomes of other cell-types and tissues have recently been under increasing scrutiny. A large study on sex differences in the transcriptome of peripheral blood in 5,241 samples showed that 3.1–13.7% of the tested genes show a sex bias in expression^21^. Two studies on sex differences in the liver transcriptome reported 844 and 1,249 sex-biased genes, respectively^22,23^. These numbers are in the same order of magnitude as what we report here for endothelial cells, highlighting that sex influences the transcriptome over variety of tissues, as underlined by work in the Genotype-Tissue Expression project^6,7^. Furthermore, sex differences in expression might also underlie differences between the sexes in response to stimuli or disease. For example, a differential transcriptomic response between the sexes has been shown in ECs undergoing shear stress^4^. In addition, a recent study on sex differences in the transcriptomic response in the myocardium to ischemia showed many interactions for gene expression between sex and ischemia^24^. Differences at baseline between the sexes should be followed up on by studies investigating sex-differential responses.

Sex steroidal influences on the endothelium have been described before^25,26^, and are associated with sex differences in disease bias. Interestingly, gene expression regulators in the list of genes with an acquired sex difference are predicted to be tightly associated with the androgen receptor (AR) (Fig. 5C), a gene expression regulator subject to sex steroids. A strong link to the AR according to our network analysis is *NR3C2*, the mineralocorticoid receptor. Recently, it has been shown that multiple transcription factors are sex-biasedly conserved over evolution^27^, among which *NR3C2*, and the AR might play a role in this bias. *AR* is not differentially expressed in adult HAECs, and neither are the estrogen receptors *ESR1, ESR2* and *GPER1*, indicating that differential RNA expression of sex steroids receptors is not directly implicated in mediating sex differences, but perhaps their interactions/interactors, location in the regulatory gene networks and/or protein levels are. The genes that have acquired a difference over time between sexes are less likely to be sex chromosomal, than those that are intrinsically different. The majority of the acquired sex differences are highly likely to be cell-type specific, since the effects of sex steroids are different per cell-type^28^. Even though sex hormonal responses were enriched in our acquired sex differences gene list, these are undoubtedly not the only cause. Gender differences are likely to play a role in the acquired differences. The majority of the intrinsically differentially expressed genes between males and females in ECs are sex chromosomal (24 out of 48), for which sex-stratified research is lacking. Unexpectedly, we found a non-random enrichment of CAD GWAS targets in the intrinsic sex difference gene set, pointing towards potential EC targets for sex differences in CAD, such as *SMG6, CELSR2, PLPP3*, and* ATP2B1.* As an example, lower *ATP2B1* expression by gene silencing has recently been shown to increase nitric oxide production and eNOS activity in HUVECs^29^. Since *ATP2B1* is expressed on lower levels in our female ECs, this might play a role in keeping ECs healthier in females by higher basal NO production, which has already been shown in rat aortas^30^, and lower ROS production, as shown in HUVECs^3^. A higher enrichment for CAD GWAS targets in the intrinsic gene set as compared to the acquired gene set also suggests that disease bias between the sexes might already be present from birth onwards, and that this is not only driven by sex steroids (changes over lifespan) and gender differences. It is important to note however, that efforts to link genetic variation on the X chromosome to CAD showed no assocations^31^, even though most of the intrinsic sex differences that we found are sex chromosomal. Intrinsic sex differences in transcriptional output of the autosome can be driven by the sex-specific effect of genetic variation^32^, sex-specific epigenetic influences, or differences in the transcriptional machinery, such as transcription factors or other cofactors. While intrinsic differences are probably not detrimental to development of males and females, in fact probably selected for, they might predispose to a variety of diseases. For example, the X-chromosomal *KDM6A*, a gene with intrinsic sex differences, protects against bladder cancer in females^33^. A recent study discovered that an XX-chromosomal complement promotes atherosclerosis in mice, by increasing the bioavailability of dietary fat^34^. It has also been shown that the Y-chromosome, possibly through the *KDM6A* Y-chromosomal counterpart *UTY*, plays a role in the proatherosclerotic reprogramming of the transcriptome^35^. The sex chromosomal intrinsic genes are probably differentially expressed between the sexes in most cell-types, as also seen in tissues^36^, but the genes are thought to have tissue-specific functions.

Even though 144 genes overlapped from the 1,135 genes higher expressed in males at birth and 845 higher expressed in males in adulthood, a strong pattern for similar Gene Ontology enrichment can be appreciated (Fig. 4C). This also holds true for the genes higher expressed in females. The same processes seem to be enriched in differential genes at the two stages, but with mostly different genes (Suppl. Fig. 4). These differential, but consistent Gene Ontology enrichments potentially underlie the functional sex differences in ECs that have been described for migration and proliferation before^3^. A strong enrichment for ribosomal protein transcripts and other translational machinery was present in genes higher expressed in males in both stages, perhaps indicating a higher protein turnover, cell size and growth or proliferation rate in male cells, since these are scenarios where more ribosomes would be needed^37^. This was coincident with a very significant signal from *MYC* as upstream regulator. *MYC* is differentially expressed as well in the boy–girl twin comparison (adjusted *p* = 0.001, log2 fold change = 0.5, higher expressed in girls). Sex differences in the levels of ribosomal proteins have been recently described in ECs^38^, but potential different mechanisms in core output from the DNA have not. The signal observed might be caused by differential responses to components of cell medium, since ribosomal biogenesis is linked to environmental and intracellular conditions^39^. However, cell culture leads to homogenization of the behaviour of cells, and therefore possibly diminishes the differences between male and female cells. Liver sinusoidal endothelial cells lose their phenotype within 3 days of monolayer culturing^40^, hence, in vivo sex differences might be more pronounced. Nevertheless, we show that a boy–girl twin model is superior for detecting sex differences as compared to a comparison between boy–boy twins and girl–girl twins, in an in vitro setting. mTOR signalling was also one of the consistent terms in differences between the male and female endothelium. mTOR signalling has been shown to be sex-specific and tissue-dependent in multiple rodent models^41,42^, and this might also be the case in the endothelium. Gene expression differences between males and females have been studied before in HUVEC pools^4^, however, we only saw overlap of the intrinsic sex chromosomal genes between our study and the one previously published. The majority of sex differences might be masked in the previously published study by not being able to correct for the effect of the environment, explaining why we found a larger number of sex-differential genes.

### Concluding remarks

In conclusion, we described and annotated sex differences in the human endothelial transcriptome at birth and in an adult setting. We showed that a boy–girl twin model is superior in detecting sex differences to a boy–boy/girl–girl twin model. In addition, endothelial gene expression is significantly different between males and females at different life stages. Some of these are consistent over life, while others are acquired, highlighting that EC studies should take sex and its interaction with lifespan into account. Annotating both gene sets with data from multiple genome-wide association studies (GWAS) revealed that genes with an intrinsic sex difference in ECs are enriched for coronary artery disease GWAS hits. This study underscores the need for treating sex as a biological variable.

### Limitations of the study

The main limitation of our study is that we used ECs from different sources (aortic and umbilical vein) for comparisons, since ECs from different sources over the human body have distinct transcriptomes^43^. Also, the sample size of the analysis involving the twins may appear as low or unbalanced as compared to the numbers used for the adult analyses. However, as the comparisons were made between twins who grew in identical environments, we hypothesized that this better powered comparison would allow us to use a small set of twins as compared to the adults in whom the variation was much larger. Indeed, the greatest statistical power is achieved in RNA-sequencing experiments in paired designs as compared to unpaired designs, since confounding at the level of all subjects can be estimated out^44^. To study this further, we performed our own study in comparing the twins to the boy–boy versus girl–girl twin design, in which the first was better powered to detect sex differences (Fig. 2A). Nevertheless, future studies with more twins might find sex differences that are even less pronounced. Since the HAECs are from male and female individuals, and therefore lack a paired design, we miss out on power to detect sex differences in adults. We tried to address this caveat by using a larger sample size in our unpaired adult design. Within the adults, we might have missed some of the sex differences due to overrepresentation of male samples. Nevertheless, in both the twins and adults we were able to identify sex differences that we feel are robust and prevalent. Lastly, we cannot distinguish sex differential effects of cell medium components (e.g. phenol-red) on the genes under study, or the sex differential gene expression in vivo from in vitro.

## Materials and methods

### Endothelial cells twins

Isolation of ECs from 28 umbilical cords of 14 twin pregnancies was performed in the UMC Utrecht. ECs from 7 boy–girl twins, 3 boy–boy twins and 4 girl–girl twins were isolated (isolation protocol and characterization in Suppl. Fig. 1). Umbilical cords were biobanked (TCBio 18-234) under the residual material ruling of the hospital (https://www.umcutrecht.nl/nl/ziekenhuis/gebruik-restmateriaal-medische-gegevens), informed consent was waived due to the fact that the material was derived anonymously. Patients or individuals can opt-out if they do not want their material to be used, which was not the case for the current study. Ethical approval of the study was given by Biobank Committee under responsibility of the Medical Ethical Committee of the UMC Utrecht. The study was performed in accordance with the Declaration of Helsinki.

### EC isolation

Umbilical cords were cut from fresh placentas and washed in 1 × PBS in petridishes, disinfected with 70% ethanol, and washed with 1 × PBS again. The vein was located, opened up with tweezers, and cannulated with a tie-wrap tied three-way tap. Next, the vein was gently flushed with 50 ml sterile 1 × PBS to remove blood clots. Then, the air from a full 50 ml syringe was pushed through the vein. To detach the ECs, the three-way tap was shut and the other end of the vein was closed with a forceps, after filling it with sterile Accutase (Innovative Cell Technologies #AT-104) until it was under tension. The vein was incubated for 5 min in a 37 °C sterile 1 × PBS filled zip lock bag and subsequently massaged for 1 min. Next, the umbilical cord was emptied over a 50 ml tube by flushing 20 ml of EGM2 (Lonza, #CC-3162) + PenStrep (Gibco Life Technologies, #15140-122) through the vein. The 50 ml tube was centrifuged for 5 min at 330×*g* RT. Pellet was resuspended in medium, diluted 1:1 with freezing medium (20% DMSO (Sigma Life Sciences #D2650) in EGM2 medium + PenStrep) and frozen down. To remove remaining blood, cells were thawed when needed, plated and cultured at 37 °C 5% CO_2_ in EGM2 + PenStrep until confluent prior to RNA isolation.

### Endothelial cells adults

Human aortic endothelial cells (HAEC) were isolated from aortic explants of 43 female and 129 male adult heart transplant donors in the UCLA transplant program and grown to confluence in 100 mm dishes as described previously^45^. All protocols involving humans were approved by UCLA Institutional Review Board. Gene expression profiles of the human cells were determined using the Affymetrix HT HG-U133A microarray, which contains 18,630 probes. Intensity values were normalized with the robust multiarray average normalization method implemented in the affy package in Bioconductor. Expression data are available in Gene Expression Omnibus accession GSE30169.

### RNA isolation

RNA was isolated from confluent HUVECs with the NucleoSpin RNA kit (Macherey–Nagel, Germany) according to the manufacturer’s protocol. RNA quantity and quality was checked with the Xpose (Trinean, Belgium). RNA integrity numbers were checked by LC Sciences (Houston, TX, USA) with a Bioanalyzer (Agilent, USA).

### Flow cytometry

Vials containing HUVEC were taken from the liquid nitrogen tank and thawed at 37 °C. Next, cells were transferred to a 50 ml tube, using 15 ml of FACs buffer, containing 0.2% EDTA (ASC Reagent, #101448118), 5% heat-inactivated (HI) FBS (BIOWEST, #S1810-500) in 1 × sterile PBS (Gibco Life Technologies, #10010015), which was added dropwise to the cells. To obtain single-cell suspensions, the 15 ml of HUVECs and FACs buffer were filtered using a 70-µM cell strainer (Greiner bio-one, #542070). The cell suspension was centrifuged (330×*g*, 5 min at RT) and the pellet was dissolved in 1 ml of FACs buffer. This was followed by counting the cells with an automated cell counter (Bio-Rad, TC10), using 10 µL cell suspension and 10 µL of Trypan Blue (Sigma Life Science, T8154). Next, cells were diluted to a final concentration of 1 × 10^6^ cells/ml, and this was divided into portions of 1 ml. This was followed by another centrifuging step (330×*g* for 5 min at RT), and the resulting pellet was re-suspended in 50 µL FACS buffer. Next, 100 µL of antibody mix was added to the sample. The antibody mix was prepared by mixing 5 µL of CD73 (Biolegend, Clone AD2, #303726), CD41 (Biolegend, Clone HIP8, #303726), CD146 (eBioscience, Clone P1H12, #12-1469-42), CD45 (BD Pharmingham, Clone HI30, #564,105), CD14 (Biolegend, Clone M5E2, #303814), CD31 (Biolegend, Clone V P025, #30120), 10 µL of CD105 (Bio-Rad, Clone SN6, #MCA1557A488) and 60 µL of 1 × PBS per sample. After an incubation of 30 min at 4 °C in the dark, cells were washed by adding 2 ml of 1 × PBS, and vortexed. After washing, cells were spun down (330×*g* for 5 min at RT), re-suspended in 1 ml of PBS, to which 1µL of viability antibody (Invitrogen by ThermoFisher Scientific, #65-0866-14) was added and incubated for 30 min at 4 °C in the dark. Subsequently, cells were washed with 2 ml FACs buffer, spun down (330×*g* for 5 min at RT) and re-suspended in 250 µL of FACs buffer. Samples were measured on the flow cytometer (Gallios, Beckman Coulter, Fullerton, CA, USA) and analysis of the flow cytometry data was performed using Kaluza 1.5 software (Beckman Coulter, Fullerton, CA, USA).

### Bioinformatics

#### RNA-sequencing

RNA-sequencing was performed by LC Sciences, Houston, TX, USA with PolyA library preparation, 150 base-pair paired end sequencing. The average sequencing depth was 40 million reads. Read mapping using STAR^46^ and summarization using htseq-count^47^ were performed by UMC Utrecht Bioinformatics Expertise Core.

All subsequent analyses were performed in R (v3.5.1) and RStudio (v1.1.463).

#### Differential gene expression

Differential gene expression for the RNA-sequencing data was calculated using DESeq2 (v1.22.1)^48^. The only difference for the comparisons between the same-sex twins and the boy–girl twins was that we could correct for the mother of the twin in the boy–girl twins. We corrected both models for *FOS* RPM-counts in the DESeq call, as some of the ECs seemed activated. Genes were called differentially expressed if FDR < 0.1. Differential gene expression for the microarray data was performed using limma (v3.38.3)^49^. Genes were called differentially expressed if *p*-value < 0.05. We used a less stringent cut-off as compared to the HUVECs, as this was an unpaired analysis. Since the microarray was limited to genes that were probed, we only used the genes that were present on the microarray for the RNA-seq analyses as well. The *p*-value for overlap between the sets was calculated with a hypergeometric test in R. The definition for the intrinsic gene set was: adjusted *p*-value < 0.1 at birth and *p*-value < 0.05 in adults. The definition for the acquired gene set was: *p*-value > 0.5 at birth and *p*-value < 0.05 in adults, as well as an absolute log-fold change more extreme than the median log-fold change of the genes with *p*-value < 0.05 for stringency.

#### Gene enrichments

Gene-sets from the Hallmark lists were used to calculate gene enrichments within R^50^ using hypergeometric tests, with a population size of 20,000. Gene ratios and *p-*values for all tests can be found in the Suppl. Data File. Radar plots were generated with the fmsb package. Gene ontology analysis for Fig. 4C was performed and visualized using clusterProfiler (v3.10.0)^51^. Upstream regulator analysis was performed by using Ingenuity Pathway Analysis (Qiagen, Germany). Sex-hormonal terms for estrogens (Hallmarks), androgens (Hallmarks), and progestin (M2191 MSigDB) were combined into one major set (Suppl. Data File) to determine enrichment for sex-hormonal targets.

#### Evolutionary conservation

PhastCon scores were calculated with the GenomicScores package^52^ and the phastCons100way.UCSC.hg19 package^53^ in R, and summarized by taking the mean of the PhastCon scores of all the bases of either the promoter (200 bp upstream of the transcription start site to 100 bp downstream of the transcription start site) or the entire gene, as determined by the TxDb.Hsapiens.UCSC.hg19.knownGene package.

#### StringDB analyses

To determine potential associated regulators that would be missed by using transcriptomic data alone, a list of transcription factors and epigenetic modifiers was constructed using EpiFactors^54^ and a recent review on the human transcription factors^55^. The transcription factors and epigenetic modifiers of the intrinsic and the acquired gene set were then entered into String^56^ to visualize and analyze associations, as well as adding the top 5 most connected associations to our input with the “More”-button. Three Y-chromosomal genes (intrinsic) could not be found in StringDB (*ZFY*, *UTY*, *KDM5D*).

#### Individual GWAS

GWAS targets for coronary artery disease (CAD)^11^, inflammatory bowel disease^12^, Crohn’s disease^12^, ulcerative colitis^12^, Alzheimer’s disease^13^, body mass index^14^, heel bone mineral density^15^, height^16^, hypertension^17^, multiple sclerosis^18^, rheumatoid arthritis^19^, and type 2 diabetes mellitus^20^ were acquired from the GWAS catalog under the MAPPED_GENE variable. The contents of the MAPPED_GENE variable were split on commas and hyphens in R using strsplit and subsequently used as the GWAS targets. GWAS catalog IDs and associated information can be found in the Suppl. Data File.

#### GWAS catalog

The entire GWAS catalog was downloaded on 15th June 2019. The unique list of genes within the GWAS catalog was extracted to calculate overlap with the acquired or intrinsic gene list or with random genes.

#### Permutations

All permutations (evolutionary conservation, regulator content, GWAS targets) were performed 10,000 times in R by sampling from random genes and taking the medians of their associated values. The observed comparator value was calculated as the median of a gene set, such as of the acquired or the intrinsic gene set. Permutation *p*-values were calculated by determining the amount of permuted values of random genes, that were higher or lower than the observed comparator value divided by the number of permutations.

#### Public datasets

We used previously published microarray data on sex differences in HUVECs (GSE552212)^4^ for a comparison with our datasets. The set was analysed using the standard GEO2R functions, and subsequently loaded into R. A gene was considered differentially expressed if FDR < 0.1.

## Supplementary information

Supplementary Data File.

Supplementary Information.

## Data Availability

HUVEC RNA-sequencing count tables are available upon request.

## Abbreviations

CAD: Coronary artery disease
DEG: Differentially expressed gene
EC: Endothelial cell
HAEC: Human aortic endothelial cell
HUVEC: Human umbilical vein endothelial cell
GWAS: Genome-wide association study
