# Supplementary material for: Intrinsic transcriptomic sex differences in human endothelial cells at birth and in adults are associated with coronary artery disease targets
Source: Sci Rep. 2020 Jul 23;10:12367. doi: 10.1038/s41598-020-69451-8 (PMC7378217; doi:10.1038/s41598-020-69451-8)
Supplement: Supplementary file 2 — Supplementary Information. [file 41598_2020_69451_MOESM2_ESM.docx]

**SUPPLEMENTAL INFORMATION**

**Intrinsic transcriptomic sex differences in human endothelial cells at birth and in adults are associated with coronary artery disease targets.**

Robin J.G. Hartman^1^, MSc; Daniek M.C. Kapteijn^1^, BSc; Saskia Haitjema^2^, MD PhD; Mireille N. Bekker^3^, MD PhD; Michal Mokry^1,2^, MD PhD; Gerard Pasterkamp^2^, MD PhD; Mete Civelek^4^, PhD; Hester M. den Ruijter^1^, PhD*

^1^ Laboratory of Experimental Cardiology, University Medical Center Utrecht, The Netherlands;

^2^ Laboratory of Clinical Chemistry and Haematology, University Medical Center Utrecht, The Netherlands;

^3^ Department of Obstetrics and Gynecology, University Medical Center Utrecht, Utrecht, The Netherlands;

^4^ Center for Public Health Genomics, Department of Biomedical Engineering, University of Virginia, Charlottesville, VA 22908, USA.

*Correspondence: h.m.denruijter-2@umcutrecht.nl

Division of Heart and Lungs

Department of Experimental Cardiology

University Medical Center Utrecht, Heidelberglaan 100

PO Box 85500, 3508GA Utrecht

The Netherlands

Supplemental Figures and Supplemental Figure Legends
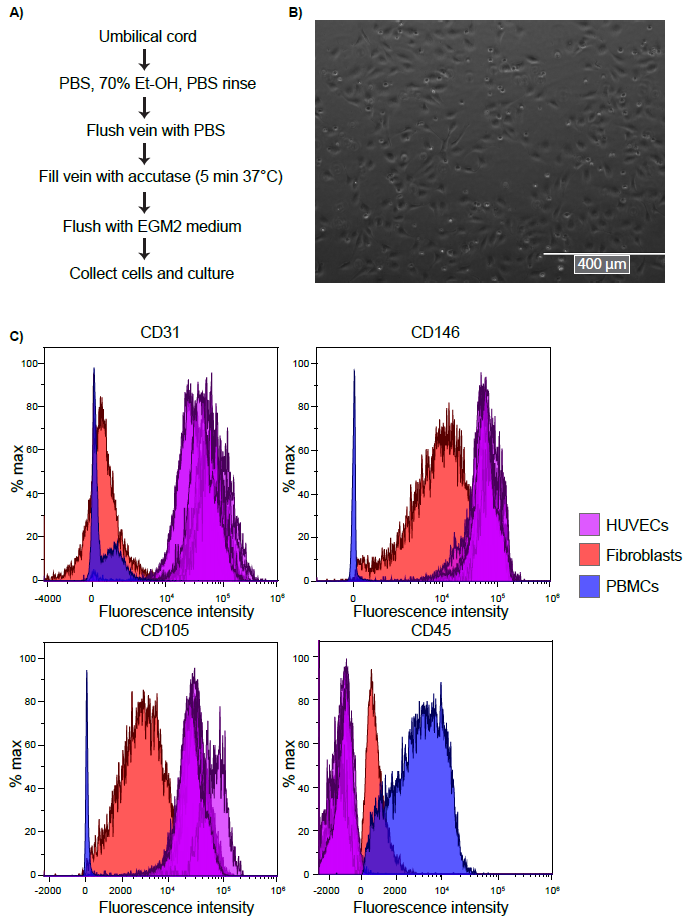


**Supplemental Figure 1. Endothelial cell isolation and characterization.** A) A simplified isolation protocol of the HUVECs is shown. B) A representative brightfield microscopy photo of the isolated HUVECs after culture is depicted. C) Fluorescence intensity densities are drawn for CD31, CD146, CD105, CD45, indicating that our isolated cells show EC markers on their membrane (CD105, CD31 and CD146 positive), and are lacking the markers that should be present on other cell types (CD45 negative).


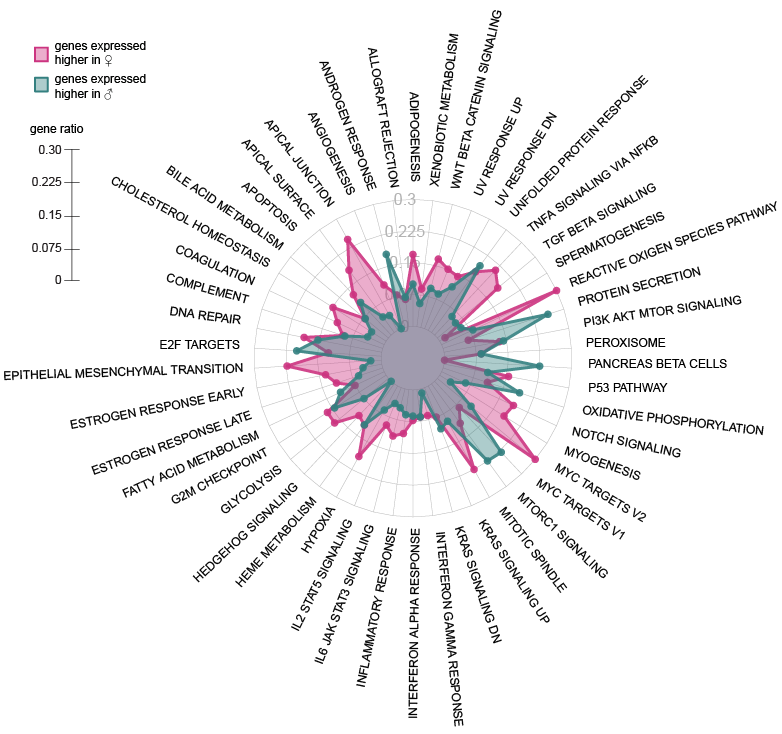
 **Supplemental Figure 2.** **Gene ratio HUVECs.** Hallmark gene enrichments are depicted in a radar plot for the genes differentially expressed between boy-girl twin HUVECs. Blue highlights the significance of the gene enrichment of genes higher expressed in males, whereas pink shows this for genes higher expressed in females. The length of the radius measures gene ratio, significance is depicted in Fig 2C.


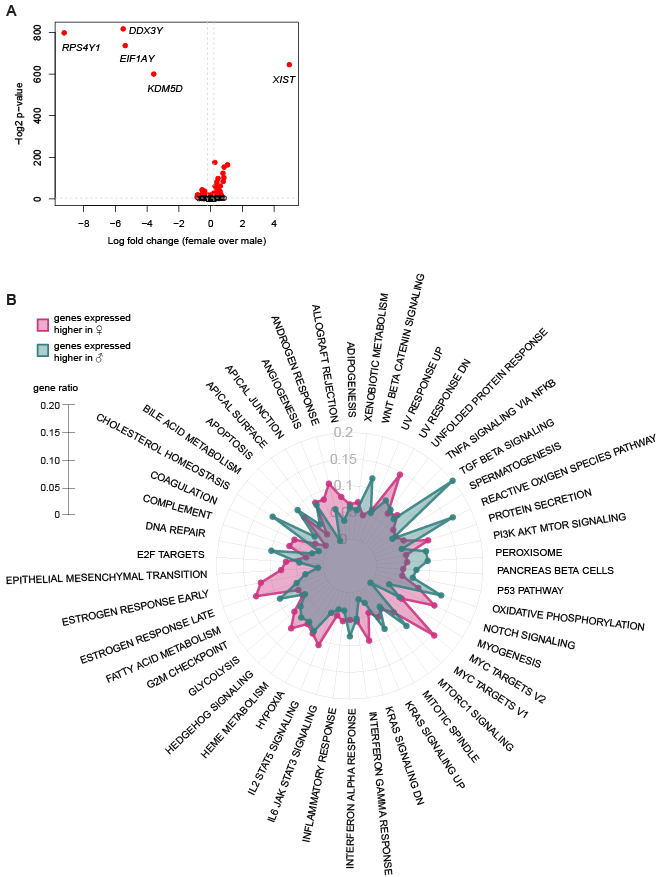


**Supplemental Figure 3. Sex differences in the adult endothelial transcriptome.** A) A volcano-plot is shown for all probes that map to non-duplicate genes.. Y-axis: –log_2_ *p*-value; x-axis: log fold change. Some typical differential sex chromosomal genes are highlighted. B) Hallmark gene enrichments are depicted in a radar plot for the genes differentially expressed between male and female HAECs. Blue highlights the significance of the gene enrichment of genes higher expressed in males, whereas pink shows this for genes higher expressed in females. The length of the radius measures gene ratio, significance is depicted in Fig 3C.


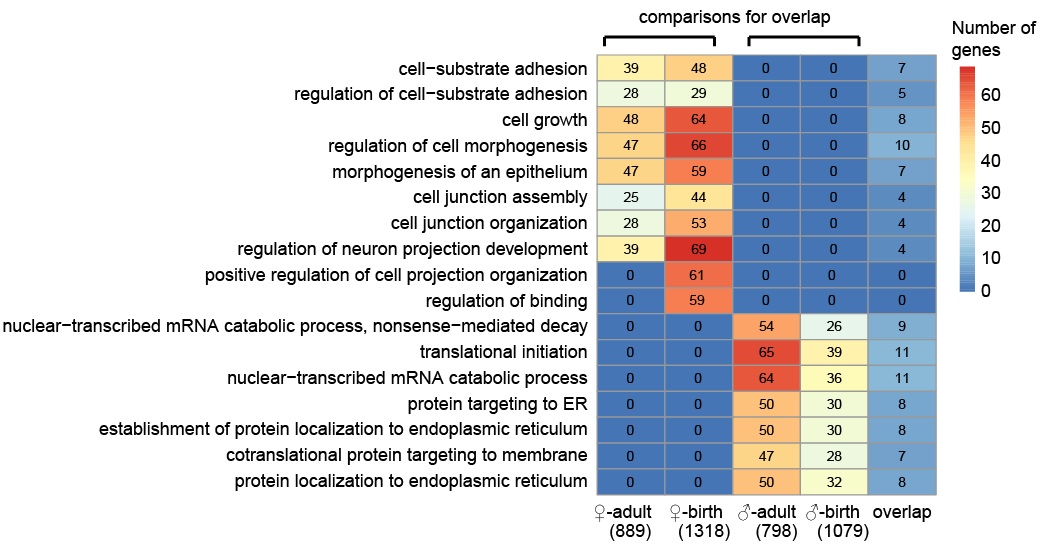


**Supplemental Figure 4. Gene Ontology overlap.** A heatmap is shown for Gene Ontology enrichments in the four different gene sets; genes higher in females in the adult stage, genes higher in females at birth, genes higher in males in the adult stage, and genes higher in males at birth, and their overlap. Terms are allocated to the rows, color indicates number of genes contributing to the Gene Ontology term. The same terms are used as Figure 4.


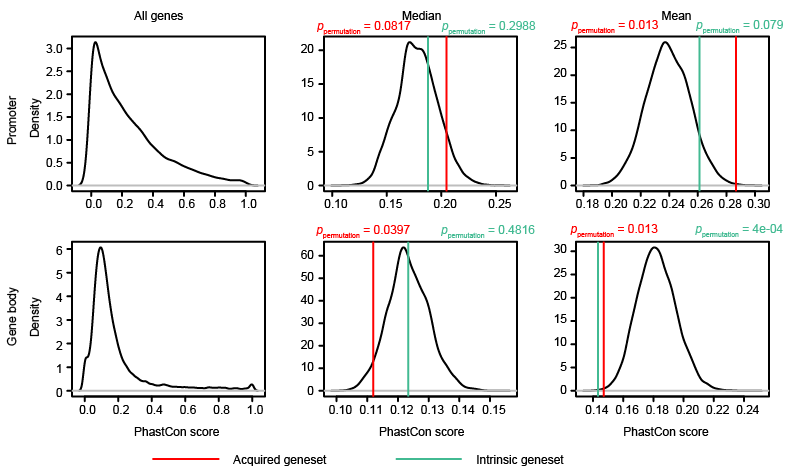


**Supplemental Figure 5. Evolutionary conservation.** Density plots of the PhastCon scores of all genes of which we interrogated the transcriptome have been plotted on the left, for either their promoter or their gene sequence. Density plots of permuted PhastCon scores are shown for promoters (top, 200bp upstream and 100bp downstream of TSS) and gene sequences (bottom), PhastCon scores have been summarized either with a median of the randomly selected genes (middle) or with a mean (right). The red line indicates the evolutionary conservation in the acquired gene set, the green line indicates this for the intrinsic gene set.


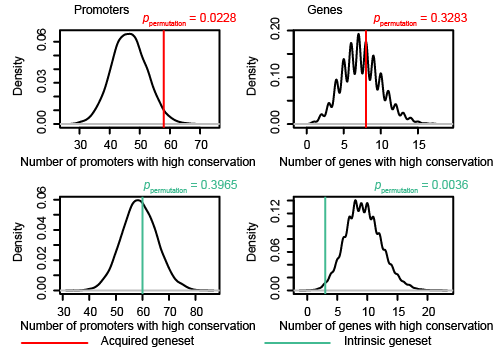


**Supplemental Figure 6. Evolutionary conservation.** Density plots of permuted and binned PhastCon scores ( > 0.4 = high) are shown for promoters (left, 200bp upstream and 100bp downstream of TSS) and gene sequences (right). The red line indicates the amount of high conservations in the acquired gene set, the green line indicates this for the intrinsic gene set.


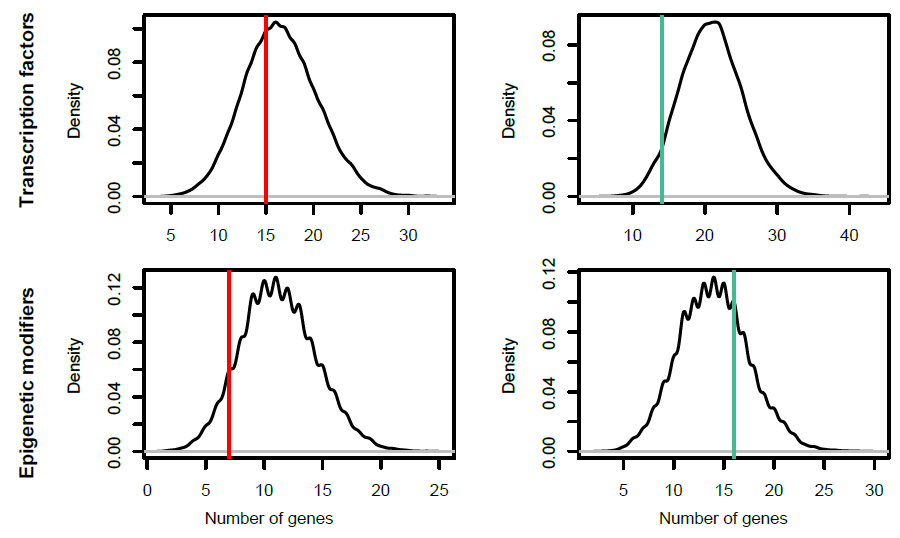


**Supplemental Figure 7. Transcription factors and epigenetic modifiers.** Density plots of permuted presence of transcription factors (top) and epigenetic modifiers (bottom) in random gene sets has been plotted. The red line indicates the amount of the respective regulators in the acquired gene set, the green line indicates this for the intrinsic gene set.


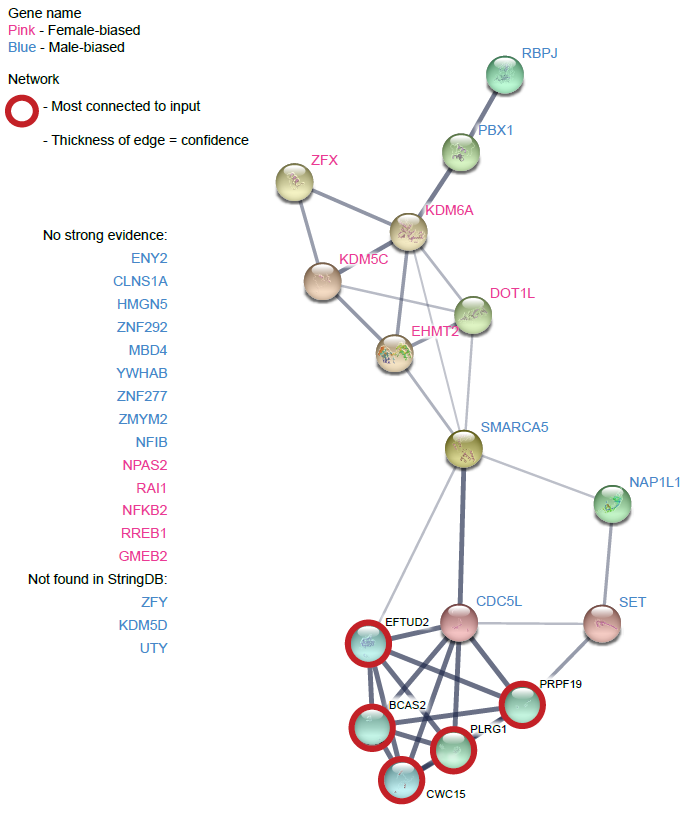


**Supplemental Figure 8.** **Intrinsic StringDB analysis.** A StringDB network is shown of transcription factors and epigenetic modifiers in the intrinsic gene set. The top 5 most connected nodes to this input have been drawn as well and are marked by a red circle. Blue letters indicate higher expression of a gene in adult male HAECs, whereas pink letters indicate higher expression in females. The thickness of the edge indicates the confidence for the association as determined by the String analysis.


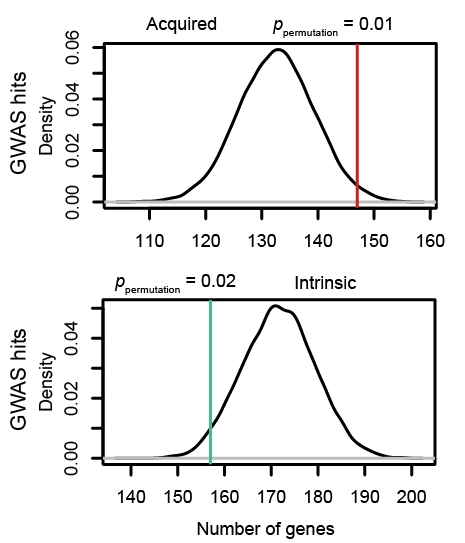


**Supplemental Figure 9. GWAS traits.** Density plots of permuted overlaps between random genes and the number of genes mapped to by a genetic variant that is associated to a trait in the GWAS catalogue. The overlap with the acquired gene set is shown on the top (*p*_permutation_ = 0.01), the intrinsic gene set is shown on the bottom (*p*_permutation_ = 0.02).
